# Supplementary material for: Whole-cell modeling of E. coli colonies enables quantification of single-cell heterogeneity in antibiotic responses
Source: PLoS Comput Biol. 2023 Jun 16;19(6):e1011232. doi: 10.1371/journal.pcbi.1011232 (PMC10310025; doi:10.1371/journal.pcbi.1011232)
Supplement: S1 Text — This file contains more detailed descriptions of the new submodels and other changes, including tables of all the new parameters used during model construction. (PDF) [file pcbi.1011232.s012.pdf]

.

Supplementary Materials for Whole-cell modeling of *E. coli* colonies enables quantification of single-cell heterogeneity in antibiotic responses

Christopher J. Skalnik [1‡], Sean Y. Cheah [1‡], Michael Y. Yang [1‡], Mattheus B. Wolff [1], Ryan K. Spangler [1^], Lee Talman [2], Jerry H. Morrison [1], Shayn M. Peirce [2], Eran Agmon [1,3], and Markus W. Covert [1*]

[1]Department of Bioengineering, Stanford University, Stanford, CA, USA
[2]Department of Biomedical Engineering, University of Virginia, Charlottesville, VA, USA
[3]Center for Cell Analysis and Modeling and Department of Molecular Biology and Biophysics, University of Connecticut Health, Farmington, CT, USA
‡These authors contributed equally to this work.
^Current Address: Altos Labs, Redwood City, California, United States

March 15, 2023

Contents

1 Introduction **3**

2 Single-cell *E. coli* model **3**
2.1 wcEcoli 3
2.2 Simulation parameters 4
2.3 Simulation state 4
2.3.1 Save state 4
2.3.2 Pseudo-random number generator seeds 5
2.4 Partitioning 5
2.5 Migration tests 6

2.6 EcoliSim 6
2.7 EngineProcess 6
2.8 Cell division 7

3 Spatial environment model **7**
3.1 Cell shape 8
3.1.1 Inputs and outputs 8
3.1.2 Model description 8
3.1.3 Parameters 9
3.2 Multibody physics 9
3.2.1 Inputs and outputs 9
3.2.2 Model description 9
3.2.3 Parameters 10
3.3 Reaction diffusion 10
3.3.1 Inputs and outputs 10
3.3.2 Model description 10
3.3.3 Parameters 11

4 Antibiotic response model **11**
4.1 Membrane permeability 11
4.1.1 Inputs and outputs 11
4.1.2 Model description 12
4.1.3 Parameters 12
4.2 Antibiotic diffusion, export, and hydrolysis 12
4.2.1 Inputs and outputs 12
4.2.2 Model description 13
4.2.3 Parameters 15
4.3 Tetracycline-induced changes to gene expression 15
4.3.1 Model description 15
4.4 Tetracycline binding to ribosomes 16
4.4.1 Inputs and outputs 16
4.4.2 Model description 16
4.4.3 Parameters 17
4.5 PBP binding and inhibition 18
4.5.1 Inputs and outputs 18
4.5.2 Model description 18
4.5.3 Parameters 19
4.6 Cell wall growth, division, and lysis 20
4.6.1 Inputs and outputs 20
4.6.2 Model description 20
4.6.3 Murein adjustment 21
4.6.4 Parameters 21

References **23**

1 Introduction

This supplementary material includes a more detailed description of the *E. coli* model used for the simulations discussed in the main text. This model, which we call vivarium-ecoli, was a fork of wcEcoli – the Covert lab's *E. coli* Whole-Cell Modeling Project [23; 36]. It recreated the wcEcoli model using Vivarium – a framework for multiscale, compositional modeling [1]. This supplement describes the operation of the original model and how it was migrated to Vivarium (section 2), the new processes that turned the model into an agent which could be plugged into a spatial environment with other whole-cell models (section 3), and finally the implementation of antibiotic response mechanisms (section 4).

2 Single-cell *E. coli* model

The single-cell model used in this paper was based on a snapshot of our wcEcoli repository (`https://github.com/CovertLab/wcEcoli`) taken on May 20, 2021. Thus, we will begin by providing an overview of the wcEcoli model (2.1) before discussing the advantages offered by the new Vivarium-based model, including greater flexibility in process parameterization (2.2), data organization (2.3), process organization (2.4), simulation configuration (2.6), and simulation organization (2.7).

2.1 wcEcoli

The wcEcoli model integrated data from a wide array of databases and published reports to calculate a set of nearly 20,000 parameters for over 10,000 equations. These equations were divided into a set of modules called processes, each of which encapsulates a distinct cellular process (e.g. mRNA transcript initiation, mRNA transcript elongation, etc.). The model was initialized with counts for all molecules in the cell and used the equations contained within its processes to calculate how these counts change over time.

There were 12 main processes in vivarium-ecoli adapted from the wcEcoli model. A full description of most of these original processes and their operation can be found in the supplementary text for the original release [23]. Since these have already been described, we only list them in the current supplement. The only exception is the chromosome structure process, which was added after the original release and will be described in an upcoming publication. The code for all processes can be found under the `ecoli.processes` directory.

These are the processes migrated from the wcEcoli repository:

1. Transcription factor binding
2. Equilibrium
3. Two-component system
4. Transcript initiation
5. Transcript elongation
6. RNA degradation
7. Polypeptide initiation
8. Polypeptide elongation

9. Protein degradation
10. Metabolism
11. Chromosome replication
12. Chromosome structure

2.2 Simulation parameters

Most simulation parameters for wcEcoli were held in an object called `sim_data`, which was generated from literature and database data by a pipeline called the parameter calculator or ParCa. Further details about the ParCa can be found in the supplementary material for Macklin et al. [23].

In wcEcoli, processes derived all their parameters from this centralized `sim_data` object. By contrast, vivarium-ecoli parameterized processes using user-supplied dictionaries, providing significantly more flexibility in terms of how parameters are sourced. Indeed, in our antibiotic simulations, while most parameters were still drawn from `sim_data`, others were loaded from JSON files (see 2.6) or calculated at run time (refer to `ecoli.library.parameters` module). To extract the relevant parameters for each process from `sim_data`, we created a new `LoadSimData` class that can be found in the `ecoli.library.sim_data` module.

2.3 Simulation state

In wcEcoli, the simulation state was stored in arrays that were designed to hold preset data types. By contrast, vivarium-ecoli uses dictionary-like internal states that impose no restrictions on the types of data that can be stored, accessed, and modified by processes.

Briefly, each node in the Vivarium simulation state was a `Store` object that held data. In a typical simulation step, processes received the current state of all connected stores, then returned a set of updates to be applied to those stores. The default file used to pre-populate stores at the start of a simulation (`data/wcecoli_t0.json`) was created by saving the initial state of a wcEcoli simulation as a JSON file. Notably, vivarium-ecoli was not restricted to this single initial state file and could be initialized with any initial state in JSON format, including states saved mid-way through a cell cycle.

2.3.1 Save state

The ability to start a simulation from any initial state was new to vivarium-ecoli. To take advantage of this feature, our colony simulations can be configured to create a JSON containing the state of all cells and their shared environment at specific time points. This saved colony state could subsequently be loaded as the initial state of a future simulation and pick up from the saved point in simulated time. Note that certain internal states (e.g. the saved solution in the flux balance analysis solver used by metabolism, the state of the stochastic simulator used by protein complexation, etc.) could not be easily accessed, saved, or reloaded, preventing perfect reconstruction from saved states. In the simulations run for the present work, this imperfect reconstruction introduced slight differences between simulations that ran for the full 26002 seconds uninterrupted (e.g. the baseline simulations) and

those that were initialized with a 11550-second saved state of a baseline simulation (e.g. the antibiotic simulations).

2.3.2 Pseudo-random number generator seeds

To ensure that simulations are reproducible, vivarium-ecoli allowed users to manually set the initial state and seed of simulations via JSON configuration files or command-line arguments (see 2.6 for more information on methods of simulation configuration). All simulations initialized with the same initial state and seed will generate the same results.

If the initial state contains only a single cell, the simulation seed was used to generate separate seeds for every pseudo-random number generator (PRNG) employed by that cell (most processes have their own PRNG). If the initial state contains multiple cells, the simulation seed was first converted to a series of seeds, one per cell, each of which was then used as described in the single-cell case. Refer to `ecoli.library.sim_data` for the exact details of PRNG seeding.

Our model relied on PRNGs both for creating internal unique identifiers (IDs) and to model biological phenomena such as stochastic processes. Thus, to prevent the clashing of unique IDs after division, daughter cells were each assigned new simulation seeds by a PRNG in the mother cell. Similarly, when loading a saved state, an algorithm was used to avoid initializing the new simulation with the same seed as the simulation which generated the saved state (refer to the `run_simulation` method of `ecoli.composites.ecoli_engine_process`).

2.4 Partitioning

Since the processes in our model share molecular resources, wcEcoli used a partitioning system to prevent "overdrafts" whereby processes collectively consume more resources than are present. Partitioning occurs at every time step as follows:

1. Each process reads the currently available molecule counts and requests counts based on what is available (`calculate_request`).
2. The `Allocator` attempts to fulfill requests based on process priorities (see below).
3. Each process calculates a change in molecule counts based on the counts it was allocated (`evolve_state`).

Most processes had equivalent neutral priority with the following exceptions: protein and RNA degradation had equally higher priorities, the two-component system process had lower priority, and transcription factor binding had the lowest priority. Chromosome structure and metabolism were the only processes that were not partitioned in the base model. Instead, they ran in succession after all other processes had finished updating the simulation state. Requests of higher-priority processes were always handled before the requests of lower-priority processes. When n processes of the same priority had requests $r_1, r_2, ..., r_n$ whose sum was greater than the unallocated count c_u of a molecule, the `Allocator` allocated c_i molecules

to process i as follows, with random allocation of remainders caused by the floor function:

$$c_i = \left\lfloor c_u \cdot \frac{r_i}{\sum_{j=1}^{n} r_j} \right\rfloor$$

In this way, even if all processes were to deplete their entire share of allocated molecules, the total count would remain non-negative, preventing overdrafts.

`PartitionedProcess` was the base class for Vivarium processes that were subject to molecular partitioning in wcEcoli. This class had `calculate_request` and `evolve_state` methods. Each partitioned process is wrapped with one `Requester` process and one `Evolver` process. `Requester` processes called the `calculate_request` of the wrapped process in the first step of the scheme above. `Evolver` processes called `evolve_state` of their wrapped process in the third step.

2.5 Migration tests

To facilitate accurate migration of wcEcoli to the Vivarium framework, we developed a system for comparing each vivarium-ecoli process with its wcEcoli equivalent, ensuring that they function the same under a variety of conditions. This system included both unit tests and whole-model comparisons. Scripts containing migration tests for each process were placed in the `migration` directory. These tests were and continue to be run on every change made on the vivarium-ecoli repository.

2.6 EcoliSim

Vivarium-ecoli introduced a JSON-based interface for simulation configuration called `EcoliSim`, enabling the creation of customized configurations for different simulation runs. Using `EcoliSim`, simulations could be configured to add or remove processes from the base model, pass alternative parameters into given processes, and set the run-order of designated order-dependent processes (e.g. `Metabolism`), among other features. Configuration files could be written to "inherit" settings from other configuration files, or merged programmatically with other configurations using the `EcoliSim` interface. This allowed simulations to be built in a modular fashion, for example by combining several configurations that each add a few related processes and their associated parameters onto the base model.

2.7 EngineProcess

`EngineProcess` was a new Vivarium process created to improve the communication overhead of colony-scale simulations. This process served as a wrapper around an entire single-cell vivarium-ecoli model, allowing the Vivarium engine to assign an operating system (OS) process to each cell instead of each process within each cell. (Note that OS processes are distinct though conceptually similar to Vivarium processes.) The *E. coli* model had over a dozen processes that were required to communicate with one another for proper function (see 2.4). This communication was much faster when the processes could share memory instead of passing messages between OS processes. Thus, by packaging all the processes

of individual cells within `EngineProcess` instances, we benefitted from fast communication between processes in the same cell without sacrificing the advantages of multiprocessing for multi-cell simulations. As of writing, there is already work underway to integrate this feature seamlessly into a future version of the Vivarium core software.

2.8 Cell division

Prior work proposed that *E. coli* may control cell size by elongating an approximately constant amount per cell cycle [5]. In accordance with this, each cell in our model initiated division upon reaching a dry mass equal to its initial dry mass plus a media-specific expected dry mass increase fitted by the parameter calculator (see 2.2). Noise was introduced by scaling this fitted dry mass increase by a factor randomly sampled from $\mathcal{N}(1, 0.1)$. In the rare case that the dry mass threshold was reached before the cell has accumulated at least two complete copies of its chromosome, division was delayed until chromosome replication had completed.

During division, all cell processes and stores were duplicated, resulting in one of each per daughter cell. Store values were divided as follows:

- Bulk molecules: Each molecule went to each daughter cell with equal probability.
- Chromosomes: Each chromosome was assigned to a daughter cell with equal probability while ensuring that each daughter got at least one full chromosome.
- Promoters: Followed their associated chromosomes.
- Chromosome domains: Followed their associated chromosomes.
- Origins of replication (oriCs): Followed their associated chromosomes.
- DnaA boxes: Followed their associated chromosomes.
- Active replisomes: Followed their associated chromosomes.
- Active RNAPs: Followed their associated chromosomes.
- Incomplete RNAs: Followed their associated active RNAPs.
- Complete RNAs: Divided like bulk molecules.
- Active ribosomes: Followed their associated incomplete RNAs.
- Listeners: Daughter cells inherited most of their listener values unaltered from the mother cell. However, for some variables, such as mass, we cut their values in half upon division as an initial estimate and re-computed their actual values in the first time step post-division.

For simplicity, division happens instantaneously and the two resulting daughters are placed end-to-end in the spatial environment model.

3 Spatial environment model

The model represented the environment as a two-dimensional rectangular space. Agent locations were continuous coordinates (x, y) within this space, and each agent had an angle from the x-axis, length, width, thrust, torque, and mass. The environment was discretized into a lattice of sites, each with the same volume. The sites were larger than the agents

to reflect how even strong concentration gradients yield approximately equal concentrations over the small distances bacteria span [24]. Each site in the lattice had a concentration for each molecule in the environment.

Importantly, the agents did not interact directly; instead, all of their interactions were mediated by changes in the environment, where agents were buffeted by physical forces, molecules diffused toward homogeneity, and the media could shift between environmental conditions.

3.1 Cell shape

3.1.1 Inputs and outputs

Inputs:

- Width (constant)
- Cell volume

Outputs:

- Length
- Outer membrane surface area
- Inner membrane surface area
- Periplasm volume
- Cytoplasm volume

3.1.2 Model description

The physical shape of the cell was modeled by the newly introduced `Shape` process. During our simulations, the `MassListener` process periodically updated total cell volume by dividing current cell mass by the assumed constant density of 1.1 g/mL [2]. `Shape` then used the calculated volume to compute cell dimensions by assuming a capsule shape formed by a cylinder capped by hemispheres at each end. The process also assumed a constant width w, so cells grew exclusively by elongation [25]. Using this information, `Shape` computed the length l and outer surface area a_o of the cell as follows:

$$l = \frac{v - \frac{4}{3}\pi\left(\frac{w}{2}\right)^3}{\pi\left(\frac{w}{2}\right)^2} + w$$

$$a_o = 4\pi\left(\frac{w}{2}\right)^2 + 2\pi\frac{w}{2}(l - w)$$

The process also used a parameter f_p, the fraction of the cell's volume consumed by the periplasm, to calculate the volume of the periplasm v_p, the volume of the cytoplasm v_c, and the surface area of the inner membrane a_i:

$$a_i = a_o(1 - f_p)^{\frac{2}{3}}$$

$$v_p = v * f_p$$

$$v_c = v * (1 - f_p)$$

3.1.3 Parameters

Parameter	Value	Description
f_p	2×10^{-1}	Fraction of cell volume consumed by the periplasm [34].
w	1 µm	Width of the cell (constant) [38].

Table SM2: Parameters for the shape process.

3.2 Multibody physics

3.2.1 Inputs and outputs

Inputs:

- Location
- Length
- Width (constant)
- Angle
- Mass
- Thrust
- Torque

Outputs:

- Location
- Length
- Angle
- Mass
- Thrust
- Torque

3.2.2 Model description

The `Multibody` process was a wrapper around the physics engine pymunk (`http://www.pymunk.org/`), which can model individual cell agents as capsule-shaped rigid bodies that can move, grow, and collide. This engine was configured with elasticity (0.9) to simulate damped bacterial collisions, random jitter to model Brownian motion, and friction (0.9) to model cell-cell adhesion. For more information on the meaning of the elasticity and friction parameters, see the pymunk documentation. `Multibody` ran pymunk with a time step one-tenth of its own two-second time step to simulate the movement of agents. It then updated each agent's location, angle, thrust, and torque. Upon division, the resulting daughter cells were placed end-to-end in the same orientation as the mother.

To simulate the low Reynolds environment bacteria experience [29], the process multiplied linear forces by d_l and angular forces by d_a every pymunk time step. Since $d_l, d_a < 1$,

this multiplication mimicked the high drag of a low Reynolds number environment. Refer to Agmon et al. [1] for more details about the parameters chosen for this submodel.

3.2.3 Parameters

Parameter	Value	Description
f_j	1×10^{-4} pN	A random force applied to each agent to simulate Brownian motion. Manually tuned to recapitulate the behavior described in [30].
agent shape	segment	Assumed shape of each cell. Segments are cylinders capped with hemispheres.
(x_b, y_b)	$(50\ \mu\text{m}, 50\ \mu\text{m})$	Dimensions of the environment.
d_l	5×10^{-1}	A fraction by which linear velocities are multiplied to approximate a low Reynolds number (manually tuned).
d_a	8×10^{-1}	A fraction by which angular velocities are multiplied to approximate a low Reynolds number (manually tuned).

Table SM3: Parameters for the multibody physics process.

3.3 Reaction diffusion

3.3.1 Inputs and outputs

Inputs:

- Environmental concentrations
- For each agent:
  - Location
  - Molecules to exchange with environment

Outputs:

- Environmental concentrations
- For each agent:
  - External environment view
  - Molecules to exchange with environment

3.3.2 Model description

The `ReactionDiffusion` process simulated bounded two-dimensional fields of molecular concentrations. Each lattice site (x, y) held the local concentrations of any number of molecules, and diffusion simulated how they homogenized across local sites. At the beginning of each time step, before diffusion between sites was calculated, a set of user-defined,

enzyme-catalyzed reactions could be simulated using Michaelis-Menten kinetics. The process assumed a Hill coefficient of $n = 1$ for all reactions. This was an appropriate assumption for the AmpC-catalyzed hydrolysis of ampicillin [26], which is described in 4.2.2.

Once reaction updates were applied, the process simulated diffusion by convolving (with reflection at the edges) the 2D Laplacian kernel over the lattice (C), multiplying by the diffusion constant D and the time step Δt, and adding the result to the lattice:

$$C \leftarrow C + D\Delta t \cdot C * \begin{pmatrix} 0 & 1 & 0 \\ 1 & -4 & 1 \\ 0 & 1 & 0 \end{pmatrix}$$

Each agent could uptake and secrete molecules at its position in the lattice. The model used the `LocalField` process to convert molecular exchanges between agents and the environment into concentration deltas that were applied at each agent's lattice site.

3.3.3 Parameters

Parameter	Value	Description
(x_b, y_b)	$(50\ \mu m, 50\ \mu m)$	Dimensions of the environment.
(n_x, n_y)	$(10, 10)$	Number of bins into which each environmental dimension is discretized.
z_b	$3 \times 10^3\ \mu m$	Depth of the environment.
D	$6 \times 10^2\ \mu m^2/s$	Diffusion constant of glucose, which we use for all molecules [35].
$reaction$	amp. $\xrightarrow{AmpC}$ amp. hydro.	Environmental chemical reaction. In this case, ampicillin hydrolysis by AmpC.
$k_{cat,h,amp}$	6.5 1/s	Rate constant for ampicillin hydrolysis [26].
$K_{M,h,amp}$	9×10^{-4} mM	Michaelis constant for ampicillin hydrolysis [26].

Table SM4: Parameters for the reaction diffusion process.

4 Antibiotic response model

This section describes all the new cellular processes that were added to model the effects of tetracycline and ampicillin exposure.

4.1 Membrane permeability

4.1.1 Inputs and outputs

Inputs:

- Porin counts
- Outer membrane surface area

Outputs:

- Permeabilities to tetracycline and ampicillin

4.1.2 Model description

The ease with which a molecule is able to diffuse across a membrane can be quantified as its permeability coefficient or permeability. The `Permeability` process computed permeabilities for tetracycline and ampicillin based on the abundance of OmpF, the porin primarily responsible for tetracycline [37] and ampicillin [21] trans-membrane diffusion.

Tetracycline can cross the outer membrane either through a porin, with permeability $P_{outer,ompf,tet}$ per unit of porin concentration in the membrane, or through the inner phospholipid bilayer with permeability $P_{outer,bilayer,tet}$. With a porin count n_{ompf} and outer membrane surface area of a_o, the permeability for tetracycline crossing the outer membrane was calculated as:

$$P_{outer,tet} = \frac{n_{ompf}}{a_o} P_{outer,ompf,tet} + P_{outer,bilayer,tet}$$

Tetracycline can also cross the inner membrane, but it does not do so through porins. Since the permeability of the inner membrane to tetracycline is constant, it was not computed by the permeability process.

Ampicillin primarily enters cells through porins, not by traversing the phospholipid bilayer [21]. Its permeability through the outer membrane was computed as:

$$P_{outer,amp} = \frac{n_{ompf}}{a_o} P_{outer,ompf,amp}$$

4.1.3 Parameters

Parameter	Value	Description
$P_{outer,ompf,amp}$	6.63×10^{-1} cm·µm^2/s/porin	Permeability of outer membrane to ampicillin [21] divided by the average simulated number of OmpF porins per square micron of the outer membrane.
$P_{outer,ompf,tet}$	2.35×10^{-9} cm·µm^2/s/porin	Porin-attributable permeability of outer membrane to tetracycline [37] divided by the average simulated number of OmpF porins per square micron of the outer membrane.
$P_{outer,bilayer,tet}$	7×10^{-8} cm/sec	Permeability of outer membrane to tetracycline without porins [37].

Table SM5: Parameters for the permeability process.

4.2 Antibiotic diffusion, export, and hydrolysis

4.2.1 Inputs and outputs

Inputs:

- Periplasmic and cytoplasmic antibiotic concentrations
- External antibiotic concentrations
- Permeabilities

Outputs:

- Periplasmic and cytoplasmic antibiotic concentrations
- Exchange of antibiotics with environment

4.2.2 Model description

The `AntibioticTransportOdeint` process simulates trans-membrane diffusion, export, and hydrolysis.

Tetracycline diffusion across the outer membrane

Under physiological conditions, tetracycline can form univalent cations by chelating magnesium ions that are readily available at the surface of the outer membrane (OM) [27; 37]. These magnesium-tetracycline chelates (Tc-Mg^{+}) preferentially cross the outer membrane by diffusing through the OmpF porin [37]. The rate of diffusion is thought to be dependent on the Donnan potential, which is generated by negatively charged, membrane-derived oligosaccharides in the periplasm that cannot diffuse through the OM [33].

To model the diffusion of this charged species across the outer membrane, we used the Goldman-Hodgkin-Katz (GHK) flux equation:

$$J_S = P_S \frac{z_s^2 F^2 E}{RT} \frac{[S]_i - [S]_o \exp\left(\frac{-z_s F E}{RT}\right)}{1 - \exp\left(\frac{-z_s F E}{RT}\right)}, \tag{1}$$

where J_S is the current density contributed by ion S (A/m^2, positive means outward flow of positive charge), P_S is the permeability of the outer membrane for ion S (m/s), $[S]_o$ is the external concentration of ion S (mol/L), $[S]_i$ is the internal concentration of ion S (mol/L), z_s is the charge of ion S, E is the membrane potential (J/C), F is Faraday's constant (C/mol), R is the gas constant (J/K/mol), and T is the temperature (K).

The GHK flux equation assumes a constant electric field across the membrane and has previously been shown to fit biological diffusion data measured *in vitro* [10; 14; 16]. Prior mathematical analysis revealed that this "constant field" assumption holds only if the following conditions are met [22]:

- The magnitude of the net charge density in the membrane must be small
- The membrane must be thin

Specifically, for membranes on the order of 100 Å thick, including the outer membrane of *E. coli* [3; 11], the magnitude of the net charge density in the membrane must be lower than that produced by 10^{-3} M of a univalent cation in solution. Since we added tetracycline at concentrations on the order of 10^{-6} M, the constant field assumption was reasonable and the GHK flux equation could be safely applied.

To approximate the rate at which Tc-Mg^+ diffuses across the outer membrane, we performed a unit conversion on the GHK flux equation (Eq. 1) and rearranged to yield influx in the form of change in concentration of a molecule per unit time $\frac{d[S]_i}{dt}$:

$$\frac{d[S]_i}{dt} = -\frac{J_S a_o}{z_s F v_p} = \frac{a_o P_S}{v_p}\frac{z_s FE}{RT}\frac{[S]_o - [S]_i \exp\left(\frac{z_s FE}{RT}\right)}{\exp\left(\frac{z_s FE}{RT}\right) - 1}, \tag{2}$$

where v_p is the periplasmic volume and a_o is the outer membrane surface area.

Tetracycline diffusion across the inner membrane

Fick's first law of diffusion states that the rate of change in internal concentration $\frac{d[S]_i}{dt}$ of a molecule with external concentration $[S]_o$ and permeability P_S across a membrane with area A is:

$$\frac{d[S]_i}{dt} = \frac{AP_S}{V}([S]_o - [S]_i), \tag{3}$$

Since the inner membrane does not contain porins, tetracycline is believed to diffuse from the periplasm into the cytoplasm in an uncharged form [37]. This would mean that there are no electrical influences to consider, and Eq. 3 can be and was used instead of Eq. 2.

Ampicillin diffusion across the outer membrane

Ampicillin is an uncharged molecule, so its diffusion across the outer membrane was modeled using Fick's law (Eq. 3). Ampicillin diffusion across the inner membrane is minimal [21] and was not considered in our model.

Antibiotic export

Tetracycline and beta-lactams are exported from *E. coli* by efflux pumps, predominantly AcrAB-TolC [21]. Before the identification of AcrAB-TolC, Thanassi et al. [37] postulated the existence of and computed kinetic parameters for this pump with tetracycline as a substrate. Later, these kinetic parameters were measured experimentally for ampicillin [21]. The parameters used in our model have been compiled in Table SM6.

We assumed Michaelis-Menten kinetics with potential cooperativity, so the efflux rate v is:

$$v = \frac{k_{cat}[E][S]^n}{K_M + [S]^n} \tag{4}$$

In the literature, v_{max} values (nmol/mg dry mass/sec) are reported instead of k_{cat} (1/sec) for these reactions, so we computed k_{cat} as follows, where $[\bar{E}]$ is the average pump concentration, $\bar{m}$ is the average dry mass, and $\bar{v_p}$ is the average periplasmic volume (all drawn from a single-cell simulation with an initial seed of 0):

$$k_{cat} = v_{max}\frac{1}{[\bar{E}]} \cdot \frac{\bar{m}}{\bar{v_p}} \tag{5}$$

Beta-lactam hydrolysis

The *E. coli* reference genome used by our models (`https://www.ncbi.nlm.nih.gov/nuccore/U00096.3`) contains the endogenous beta-lactamase gene *ampC* that exhibits low and non-inducible expression [26]. We modeled the hydrolysis of ampicillin by AmpC as a Michaelis-Menten reaction (Eq. 4) where the hydrolysis product is inert.

Implementation

Diffusion reactions take place too quickly for the Vivarium engine to accurately integrate using the process time step of two seconds. Therefore, the `AntibioticTransportOdeint` process used the `solve_ivp` numerical integration function from SciPy [32] to simulate the ordinary differential equations (ODEs) described above over the course of each two-second time step.

4.2.3 Parameters

Parameter	Value	Description
$P_{inner,tet}$	3×10^{-6} cm/sec	Inner membrane permeability to tetracycline [37].
E	-2.15×10^{1} mV	Donnan potential across the outer membrane [33].
$[\bar{E}]$	7.16×10^{-4} mM	Average concentration of AcrAB-TolC.
$\bar{m}$	1.64×10^{3} fg	Average wet mass of the cell.
$\bar{v_p}$	2.98×10^{-1} fL	Average volume of the periplasm.
$v_{max,e,amp}$	6.9×10^{-2} nmol/mg/sec	Maximum rate of ampicillin export [21].
$K_{M,e,amp}$	2.16×10^{-3} mM	Michaelis constant for ampicillin export [21].
$n_{e,amp}$	1.9	Hill coefficient for ampicillin export [21].
$v_{max,e,tet}$	3.33×10^{-3} nmol/mg/sec	Maximum rate of tetracycline export [37].
$K_{M,e,tet}$	2×10^{-1} mM	Michaelis constant for tetracycline export [37].
$n_{e,tet}$	1	Hill coefficient for tetracycline export.
$k_{cat,h,amp}$	6.5 1/s	Rate constant for ampicillin hydrolysis [26].
$K_{M,h,amp}$	9×10^{-4} mM	Michaelis const. for ampicillin hydrolysis [26].
$n_{h,amp}$	1	Hill coefficient for ampicillin hydrolysis [26].

Table SM6: Parameters for the antibiotic diffusion, export, and hydrolysis (antibiotic transport) process.

4.3 Tetracycline-induced changes to gene expression

We modeled tetracycline-induced gene regulatory changes using the `TFBinding` process that was already in the model [23].

4.3.1 Model description

In our model, tetracycline-induced gene regulation began with the inactivation of MarR by tetracycline, which we modeled as a reversible reaction at chemical equilibrium. In the model,

as the fraction of inactive MarR increased, so did the occupation of MarA on the promoters of its downstream regulatory targets. The effect of MarA binding to promoters was tuned to yield comparable mRNA fold changes as those measured for *E. coli* cells exposed to 1.5 mg/L of tetracycline (Fig. S6B) [40]. Notably, we chose to silence MarA activity in the complete absence of MarR, preserving baseline behavior at the cost of rare ($< 5\%$) delays in tetracycline-induced gene regulation.

In addition to direct regulation by MarA, the *ompF* gene, which encodes the primary porin for tetracycline influx, is also subject to post-transcriptional regulation that is triggered by tetracycline. Specifically, MarA upregulates expression of *micF*, a small non-coding RNA that can form duplexes with *ompF* mRNA and thereby decrease *ompF* translation [9]. In the absence of experimental data, we assumed that all synthesized *micF* transcripts immediately form irreversible duplexes with free-floating *ompF* transcripts and that the resulting duplexes have the same half-life as standalone *ompF* transcripts. Additionally, we assumed that MarA increased production of *micF* RNA just enough to sequester nearly all *ompF* transcripts when cells were exposed to 1.5 mg/L of tetracycline.

4.4 Tetracycline binding to ribosomes

4.4.1 Inputs and outputs

Inputs:

- Free tetracycline concentration
- Free 30S concentration
- Free 70S concentration
- 50S concentration
- Tetracycline-bound 30S concentration
- Count of tRNAs
- Cytoplasm volume

Outputs:

- Free tetracycline concentration
- Free 30S concentration
- Free 70S concentration
- 50S concentration
- Tetracycline-bound 30S concentration

4.4.2 Model description

Tetracycline is a bacteriostatic antibiotic that inhibits protein synthesis by binding to a highly conserved pocket in the 30S ribosomal subunit near the A site, interfering with accommodation of aminoacylated tRNA during polypeptide elongation [15; 18]. Thus, we introduced the `TetracyclineRibosomeEquilibrium` process to model ribosomal binding as a competition between tetracycline and aminoacylated tRNAs. Since the binding constants

for tRNAs and tetracycline are both much smaller than the average concentration of active ribosomes ($10^{-6}M < 10^{-5}M$), we assumed that all ribosomal A sites are bound by one or the other at all times. See Table SM7 for the exact binding constants.

Specifically, given a tRNA-ribosome binding constant of K_{tRNA}, a tetracycline-ribosome binding constant of K_{Tc}, c_{tRNA} aminoacylated tRNA molecules, c_{Tc} tetracycline molecules, $c_{R,Tc}$ tetracycline-bound ribosomes, and $c_{R,tRNA}$ tRNA-bound ribosomes, we know that at equilibrium the following holds:

$$r = \frac{c_{R,Tc}}{c_{R,tRNA}} = \frac{K_{Tc} \cdot c_{Tc}}{K_{tRNA} \cdot c_{tRNA}}$$

Thus, the fraction f of ribosomal A sites bound by tetracycline at equilibrium is equal to:

$$\begin{aligned} f &= \frac{c_{R,Tc}}{c_{R,Tc} + c_{R,tRNA}} \\ &= \frac{\frac{c_{R,Tc}}{c_{R,tRNA}}}{\frac{c_{R,Tc}}{c_{R,tRNA}} + 1} \\ &= \frac{r}{r+1} \end{aligned} \tag{6}$$

At the time of writing, vivarium-ecoli lacked a proper mechanistic model for tRNA charging. Since the average cell in our model reported a total tRNA count about three times the literature consensus for charged tRNAs [12; 17], the simulated tRNA count was always multiplied by a factor of 0.33 before further calculations. Additionally, we assumed that each active ribosome had two bound tRNAs at all times (one in the E site and one in the P site), further decreasing the pool of charged tRNAs competing for A site binding.

Since we only found parameters that describe the equilibrium binding of tetracycline and tRNAs to ribosomes and not their binding kinetics, we used a root finder (`root_scalar` from Scipy [32]) to solve for the equilibrium concentrations of free tetracycline, tetracycline-bound ribosomes, and free ribosomes. The algorithm used to compute this equilibrium is described in Algorithm 1.

4.4.3 Parameters

Parameter	Value	Description
K_{tRNA}	$4.5 \times 10^6\ M^{-1}$	Association constant for tRNA-ribosome binding. Tuned so that simulations give the correct tetracycline IC50 (see Fig. S6) [31].
K_{Tc}	$3 \times 10^6\ M^{-1}$	Association constant for tetracycline-ribosome binding [13].

Table SM7: Parameters for tetracycline-ribosome binding.

Algorithm 1: Tetracycline-ribosome equilibrium

Input : $c_{tRNA,tot}$ total tRNA count
Input : c_{Tc} free cytoplasmic tetracycline count
Input : c_{70S} active ribosome count
Input : c_{30S} free small subunit count
Input : $c_{30S\text{-}Tc}$ tetracycline-bound small subunit count

1. Estimate the count of free, charged tRNAs from the total count.

$$c_{tRNA} = c_{tRNA,tot} \cdot 0.33 - 2 \cdot c_{70S}$$

2. Use root finder to calculate Δ tetracycline molecules to bind to (or unbind from) 30S ribosomes such that the following equation holds:

$$c_{30S\text{-}Tc} + \Delta = f \cdot (c_{70S} + c_{30S} + c_{30S\text{-}Tc}),$$

where f is calculated using Eq. 6 with $c_{Tc} = c_{Tc} - \Delta$.

3. Distribute the calculated count of ribosomes bound to tetracycline proportionally among 30S subunits and 70S active ribosomes.

$$c_{70S\text{-}Tc,target} = \left\lfloor (c_{30S\text{-}Tc} + \Delta) \cdot \frac{c_{70S}}{c_{70S} + c_{30S} + c_{30S\text{-}Tc}} \right\rfloor$$

$$c_{30S\text{-}Tc,target} = c_{30S,Tc} + \Delta - c_{70S\text{-}Tc,target}$$

Result: Increase/decrease c_{30S} so that $c_{30S\text{-}Tc}$ can be decreased/increased to $c_{30S\text{-}Tc,target}$. Decrease c_{70S} by $c_{70S\text{-}Tc,target}$ and increase $c_{30S\text{-}Tc}$ by $c_{70S\text{-}Tc,target}$ (inactivated 70S active ribosomes assumed to dissociate and form 30S subunits bound to tetracycline).

4.5 PBP binding and inhibition

4.5.1 Inputs and outputs

Inputs:

- Periplasmic ampicillin concentration
- Count of PBP1A, PBP1B (γ isoform) in the cell
- Count of newly produced murein

Outputs:

- Fraction of PBP1A, PBP1B not bound by ampicillin
- Allocation of newly produced murein to pools that are either usable (crosslinked) and unusable (uncrosslinked) for incorporation into the cell wall

4.5.2 Model description

We modeled the ampicillin-mediated inhibition of PBP transpeptidase activity using a Hill equation with no cooperativity. This model was implemented in the `PBPBinding` process.

We calculated the proportions of unbound, active PBP1A and PBP1B as follows:

$$\theta_{PBP1A} = \frac{1}{\left(1 + \frac{[\text{Amp}]}{K_{A,PBP1A}}\right)},$$
$$\theta_{PBP1B} = \frac{1}{\left(1 + \frac{[\text{Amp}]}{K_{A,PBP1B}}\right)},$$

where $\theta_{PBP1A}, \theta_{PBP1B}$ are the proportion of unbound PBP1A and PBP1B respectively, [Amp] is the concentration of ampicillin in the periplasm, and $K_{A,PBP1A}$ and $K_{A,PBP1B}$ are the ampicillin concentrations necessary to inhibit transpeptidation activity by 50% for PBP1A and PBP1B, respectively.

$K_{A,PBP1B}$ was parameterized directly from literature [6], whereas $K_{A,PBP1A}$ (specifically for inhibition of transpeptidation) has not been measured to our knowledge. However, ampicillin binding affinities for PBP1A and PBP1B have been measured as being within one order of magnitude [7; 19]. Assuming that the ability of ampicillin to inhibit transpeptidation is proportional to its binding affinity, $K_{A,PBP1A}$ should also be within one order of magnitude of $K_{A,PBP1B}$. Using this plausible range as a guideline, we estimated a $K_{A,PBP1A}$ for ampicillin inhibition of PBP1A transpeptidase activity that resulted in cell death as expected in single-cell simulations exposed to the 2 mg/L ampicillin (MIC).

Once the proportions of unbound PBP1A and PBP1B were determined, all the nascent murein produced by `Metabolism` in the most recent time step was divided into two pools, one for crosslinked murein and the other for murein that was not crosslinked due to inhibition of PBP-catalyzed transpeptidation by ampicillin. The count of crosslinked murein was reported to the cell wall process for incorporation while uncrosslinked murein was assumed to be permanently unusable for cell wall synthesis. If a cell had c_{PBP1A} PBP1A and c_{PBP1B} PBP1B molecules, the count of crosslinked murein c_c and uncrosslinked murein c_u was partitioned from the total new murein count c_m as follows:

$$c_c = c_m \left(\theta_{PBP1A} \cdot \frac{c_{PBP1A}}{c_{PBP1A} + c_{PBP1B}} + \theta_{PBP1B} \cdot \frac{c_{PBP1B}}{c_{PBP1A} + c_{PBP1B}} \right)$$
$$c_u = c_m - c_c$$

4.5.3 Parameters

Parameter	Value	Description
$K_{A,PBP1A}$	0.7 µM	Ampicillin concentration at which PBP1A transpeptidation activity is halved.
$K_{A,PBP1B}$	1.27 µM	Ampicillin concentration at which PBP1B transpeptidation activity is halved [6].

Table SM8: Parameters for the PBP binding process.

4.6 Cell wall growth, division, and lysis

4.6.1 Inputs and outputs

Inputs:

- Murein state (incorporated, usable, and unusable pools)
- Counts of PBP1A, PBP1B
- Fraction of PBP1A, PBP1B not bound by ampicillin
- Cell wall state: lattice, extension factor, whether the wall has cracked

Outputs:

- Updated cell wall lattice
- Extension factor above resting length
- Cracking

4.6.2 Model description

Cell wall growth was modeled as a function of cell shape, available murein, and active PBPs in the `CellWall` process. Cell wall state was represented as a 2D lattice on the surface of a cylindrical shell, in which lattice positions represented the average surface area spanned by a peptidoglycan unit. Lattice positions filled with ones represented cross-linked peptidoglycan, while lattice positions filled with zeroes corresponded to gaps where peptidoglycan was not crosslinked into the cell wall.

Because the *E. coli* sacculus is elastic primarily in the long direction of the cell, we permitted lengthwise stretching of the lattice up to the experimentally determined maximum surface increase, E_{max}. At each 10 second time step, `CellWall` first attempted to relax the lattice if excess murein and PBPs were available. If current cell dimensions provided by the `CellShape` process indicated that the cell had grown, the difference between the length of the lattice and the cell length was first expressed in terms of the number of new columns to be added.

After distributing the murein available to be incorporated (calculated by the `PBPBinding` model) uniformly at random among the columns, the content of each new column was determined by sampling several peptidoglycan strands whose lengths followed a geometric distribution with parameter p_{strand} estimated from literature data. These strands (stretches of ones) were placed end-to-end with single-position gaps (0) in between to populate each newly generated column. Then, $N_{sites} = \min(\#\text{ active PBPs}, \#\text{ new columns})$ insertion sites were chosen uniformly at random along the length of the lattice. New columns were then distributed uniformly at random among the N_{sites} insertion sites such that ≥ 1 column was inserted per site.

The `CellWall` process then inserted these newly sampled columns at their designated insertion sites, resulting in a "proposed" next cell wall state. The size of the largest hole in the proposed lattice was compared with a critical hole area for lysis from literature ($A_{\text{critical}} = \pi r_{\text{critical}}^2$). If the proposed lattice crossed this threshold, the `CellWall` process first checked to see whether the cell could be saved from lysis by stretching the cell wall

further to cover the new cell length. When attempting to stretch, the necessary extension factor was first compared with $E_{\max}$ to determine whether stretching was possible. If so, the extension factor E was increased such that the lattice covered the new size of the cell without incorporation of new murein. This alternative proposed lattice was then evaluated again for cracking, since the stretching itself increased the physical size of existing lattice defects. A summary of the steps in cell wall growth is outlined in Algorithm 2.

Once the cell wall had cracked, this was read by the `LysisInitiation` process. This process then sampled a waiting time (on the order of 3.2 minutes) from a distribution fitted to literature data, representing the time that the cell spends with the inner membrane bulging out through the cell wall. Upon reaching the end of this delay period, `Lysis` was triggered, resulting in the removal of the cell from the simulation and the spilling of internal ampicillin and beta-lactamase into the environment. These environmental changes were enacted by the `ReactionDiffusion` process (see 3.3). If a cell survived until division without lysing, the two resulting daughter cells each inherited one-half of the lattice from the mother cell.

4.6.3 Murein adjustment

In the process of creating a physical representation of the cell wall, we noted that the original release of the model produced over two times more murein than necessary to completely cover the surface area of an average cell. As such, we iteratively estimated a corrected homeostatic objective for murein that resulted in approximately no leftover murein at the start and end of a representative cell cycle (seed 0). This new objective was about 2.27 times smaller than the original value and was derived from the assumption that cells do not produce significantly more murein than necessary to maintain cell wall integrity.

4.6.4 Parameters

Parameter	Value	Description
p_{strand}	7.68×10^{-2}	Strand extension probability. Fitted assuming a geometric distribution using data from [28; 41].
r_{critical}	20 nm	Critical defect radius to initiate lysis [8].
ℓ_y	1.03 nm	Length of one peptidoglycan unit (along the direction of the strand) [42].
ℓ_x	1.4 nm	Width of a glycan strand [39].
d_x	0.6 nm	Typical resting distance between glycan strands. Estimated such that the initial lattice uses all initial murein. Together with ℓ_x, consistent with literature distances between centers of adjacent strands [4; 39].
$E_{\max}$	3	Maximum permissible expansion of the cell sacculus [20].
$\hat{t}_{\text{lysis}}$	192.8 s	Mean time to lysis after bulging begins [43].

Table SM9: Parameters for the cell wall process.

Algorithm 2: Cell wall growth

Input : m crosslinked murein count
Input : $c_{PBP1A},\ c_{PBP1B}$ PBP1A/B counts
Input : $\theta_{PBP1A},\ \theta_{PBP1B}$ fraction of PBP1A/B that is active
Input : l cell length
Input : e cell wall extension factor
Input : L cell wall lattice with y rows and x columns

1. Calculate new number of columns in cell lattice.

$$x_{new} = \frac{l}{e \cdot (l_x + d_x)}$$

2. Shrink extension factor if there is excess murein.

if $\lfloor m/y \rfloor > x_{new} - x$ **then**
  $x_{new} = x + \lfloor m/y \rfloor$
  $e_{new} = \max\left(\frac{l}{x_{new}(l_x + d_x)},\ 1\right)$

3. Calculate count of murein allocated to each new column.

$$\vec{m} = \text{multinomal}(m,\ \text{repeat}((x_{new} - x)^{-1},\ x_{new} - x))$$

4. Sample new cell wall columns.

for column = 0 **to** $x_{new} - x$ **do**
  filled = 0
  $\vec{c}$ = zero-filled array of length y (new column)
  while filled < y and filled < $\vec{m}$ [column] **do**
    strand = geom(p_{strand})
    if filled + strand > y or filled + strand > $\vec{m}$ [column] **then**
      strand = min($\vec{c}$ [column] − filled, y − filled)
      Set positions from filled to filled + strand equal to 1 in $\vec{c}$
      break
    Set positions from filled to filled + strand equal to 1 in $\vec{c}$
    filled += strand + 1

4. Calculate number of active cell wall synthesis sites.

$$c_{syn} = \min(\theta_{PBP1A} \cdot c_{PBP1A} + \theta_{PBP1B} \cdot c_{PBP1B},\ y_{new} - y)$$

5. Distribute newly generated columns among c_{syn} positions in the initial lattice.

6. Record hole sizes, try to stretch if hole too large, crack if still too large.

if max hole size > $\pi r^2_{\text{critical}}$ **then**
  $e_{new} = \frac{l}{x(l_x + d_x)}$
  if $e_{new} < E_{\max}$ **then**
    if max hole size > $\pi r^2_{\text{critical}}$ **then**
      Cell wall cracked.
    else
      Cell wall intact.
  else
    Cell wall cracked.

References

[1] Agmon, E., Spangler, R. K., Skalnik, C. J., Poole, W., Peirce, S. M., Morrison, J. H., and Covert, M. W. (2022). Vivarium: an interface and engine for integrative multiscale modeling in computational biology. *Bioinformatics*, 38(7):1972–1979.

[2] Baldwin, W. W., Myer, R., Powell, N., Anderson, E., and Koch, A. L. (1995). Buoyant density of Escherichia coli is determined solely by the osmolarity of the culture medium. *Archives of Microbiology*, 164(2):155–157.

[3] Bayer, M. (1991). Zones of membrane adhesion in the cryofixed envelope of Escherichia coli. *Journal of Structural Biology*, 107(3):268–280.

[4] Braun, V., Gnirke, H., Henning, U., and Rehn, K. (1973). Model for the Structure of the Shape-Maintaining Layer of the Escherichia coli Cell Envelope. *Journal of Bacteriology*, 114(3):1264–1270. Publisher: American Society for Microbiology.

[5] Campos, M., Surovtsev, I. V., Kato, S., Paintdakhi, A., Beltran, B., Ebmeier, S. E., and Jacobs-Wagner, C. (2014). A constant size extension drives bacterial cell size homeostasis. *Cell*, 159(6):1433–1446.

[6] Catherwood, A. C., Lloyd, A. J., Tod, J. A., Chauhan, S., Slade, S. E., Walkowiak, G. P., Galley, N. F., Punekar, A. S., Smart, K., Rea, D., Evans, N. D., Chappell, M. J., Roper, D. I., and Dowson, C. G. (2020). Substrate and Stereochemical Control of Peptidoglycan Cross-Linking by Transpeptidation by Escherichia coli PBP1B. *Journal of the American Chemical Society*, 142(11):5034–5048. Publisher: American Chemical Society.

[7] Curtis, N. A., Orr, D., Ross, G. W., and Boulton, M. G. (1979). Affinities of penicillins and cephalosporins for the penicillin-binding proteins of Escherichia coli K-12 and their antibacterial activity. *Antimicrobial Agents and Chemotherapy*, 16(5):533–539.

[8] Daly, K. E., Huang, K. C., Wingreen, N. S., and Mukhopadhyay, R. (2011). Mechanics of membrane bulging during cell-wall disruption in Gram-negative bacteria. *Physical Review E*, 83(4):041922. Publisher: American Physical Society.

[9] Delihas, N. and Forst, S. (2001). MicF : an antisense RNA gene involved in response of Escherichia coli to global stress factors 1 1Edited by D. Draper. *Journal of Molecular Biology*, 313(1):1–12.

[10] Diamond, J. M. and Harrison, S. C. (1966). The effect of membrane fixed charges on diffusion potentials and streaming potentials. *The Journal of Physiology*, 183(1):37–57.

[11] DiRienzo, J. M., Nakamura, K., and Inouye, M. (1978). The Outer Membrane Proteins of Gram-Negative Bacteria: Biosynthesis, Assembly, and Functions. *Annual Review of Biochemistry*, 47(1):481–532.

[12] Dong, H., Nilsson, L., and Kurland, C. G. (1996). Co-variation of tRNA Abundance and Codon Usage inEscherichia coliat Different Growth Rates. *Journal of Molecular Biology*, 260(5):649–663.

[13] Epe, B. and Woolley, P. (1984). The binding of 6-demethylchlortetracycline to 70S, 50S and 30S ribosomal particles: a quantitative study by fluorescence anisotropy. *The EMBO journal*, 3(1):121–126.

[14] Goldman, D. E. (1943). POTENTIAL, IMPEDANCE, AND RECTIFICATION IN MEMBRANES. *Journal of General Physiology*, 27(1):37–60.

[15] Grossman, T. H. (2016). Tetracycline Antibiotics and Resistance. *Cold Spring Harbor Perspectives in Medicine*, 6(4):a025387.

[16] Hodgkin, A. L. and Katz, B. (1949). The effect of sodium ions on the electrical activity of the giant axon of the squid. *The Journal of Physiology*, 108(1):37–77.

[17] Jakubowski, H. and Goldman, E. (1984). Quantities of individual aminoacyl-tRNA families and their turnover in Escherichia coli. *Journal of Bacteriology*, 158(3):769–776.

[18] Jenner, L., Starosta, A. L., Terry, D. S., Mikolajka, A., Filonava, L., Yusupov, M., Blanchard, S. C., Wilson, D. N., and Yusupova, G. (2013). Structural basis for potent inhibitory activity of the antibiotic tigecycline during protein synthesis. *Proceedings of the National Academy of Sciences*, 110(10):3812–3816.

[19] Kocaoglu, O. and Carlson, E. E. (2015). Profiling of β-Lactam Selectivity for Penicillin-Binding Proteins in Escherichia coli Strain DC2. *Antimicrobial Agents and Chemotherapy*, 59(5):2785–2790. Publisher: American Society for Microbiology.

[20] Koch, A. L. and Woeste, S. (1992). Elasticity of the sacculus of Escherichia coli. *Journal of Bacteriology*, 174(14):4811–4819.

[21] Kojima, S. and Nikaido, H. (2013). Permeation Rates of Penicillins Indicate that Escherichia coli Porins Function Principally as Nonspecific Channels. *Proceedings of the National Academy of Sciences*, 110(28):E2629–E2634.

[22] MacGillivray, A. and Hare, D. (1969). Applicability of Goldman's constant field assumption to biological systems. *Journal of Theoretical Biology*, 25(1):113–126.

[23] Macklin, D. N., Ahn-Horst, T. A., Choi, H., Ruggero, N. A., Carrera, J., Mason, J. C., Sun, G., Agmon, E., DeFelice, M. M., Maayan, I., Lane, K., Spangler, R. K., Gillies, T. E., Paull, M. L., Akhter, S., Bray, S. R., Weaver, D. S., Keseler, I. M., Karp, P. D., Morrison, J. H., and Covert, M. W. (2020). Simultaneous cross-evaluation of heterogeneous E. coli datasets via mechanistic simulation. *Science*, 369(6502):eaav3751.

[24] Macnab, R. M. and Koshland, D. E. (1972). The gradient-sensing mechanism in bacterial chemotaxis. *Proceedings of the National Academy of Sciences*, 69(9):2509–2512.

[25] Marr, A. G., Harvey, R. J., and Trentini, W. C. (1966). Growth and division of Escherichia coli. *Journal of Bacteriology*, 91(6):2388–2389.

[26] Mazzariol, A., Cornaglia, G., and Nikaido, H. (2000). Contributions of the AmpC β-Lactamase and the AcrAB Multidrug Efflux System in Intrinsic Resistance of *Escherichia coli* K-12 to β-Lactams. *Antimicrobial Agents and Chemotherapy*, 44(5):1387–1390.

[27] Nikaido, H. and Vaara, M. (1985). Molecular basis of bacterial outer membrane permeability. *Microbiological Reviews*, 49(1):1–32.

[28] Obermann, W. and Höltje, J. (1994). Alterations of murein structure and of penicillin-binding proteins in minicells from Escherichia coli. *Microbiology*.

[29] Purcell, E. M. (1977). Life at low Reynolds number. *American Journal of Physics*, 45(1):3–11.

[30] Saragosti, J., Silberzan, P., and Buguin, A. (2012). Modeling E. coli Tumbles by Rotational Diffusion. Implications for Chemotaxis. *PLoS ONE*, 7(4):e35412.

[31] Schilling-Bartetzko, S., Franceschi, F., Sternbach, H., and Nierhaus, K. (1992). Apparent association constants of tRNAs for the ribosomal A, P, and E sites. *Journal of Biological Chemistry*, 267(7):4693–4702.

[32] SciPy 1.0 Contributors, Virtanen, P., Gommers, R., Oliphant, T. E., Haberland, M., Reddy, T., Cournapeau, D., Burovski, E., Peterson, P., Weckesser, W., Bright, J., van der Walt, S. J., Brett, M., Wilson, J., Millman, K. J., Mayorov, N., Nelson, A. R. J., Jones, E., Kern, R., Larson, E., Carey, C. J., Polat, , Feng, Y., Moore, E. W., VanderPlas, J., Laxalde, D., Perktold, J., Cimrman, R., Henriksen, I., Quintero, E. A., Harris, C. R., Archibald, A. M., Ribeiro, A. H., Pedregosa, F., and van Mulbregt, P. (2020). SciPy 1.0: Fundamental algorithms for scientific computing in Python. *Nature Methods*, 17(3):261–272.

[33] Sen, K., Hellman, J., and Nikaido, H. (1988). Porin channels in intact cells of Escherichia coli are not affected by Donnan potentials across the outer membrane. *Journal of Biological Chemistry*, 263(3):1182–1187.

[34] Stock, J. B., Rauch, B., and Roseman, S. (1977). Periplasmic space in Salmonella typhimurium and Escherichia coli. *The Journal of Biological Chemistry*, 252(21):7850–7861.

[35] Suhaimi, H., Wang, S., and Das, D. B. (2015). Glucose diffusivity in cell culture medium. *Chemical Engineering Journal*, 269:323–327.

[36] Sun, G., Ahn-Horst, T. A., and Covert, M. W. (2021). The e. coli whole-cell modeling project. *EcoSal plus*, 9(2):eESP–0001.

[37] Thanassi, D. G., Suh, G. S., and Nikaido, H. (1995). Role of outer membrane barrier in efflux-mediated tetracycline resistance of Escherichia coli. *Journal of Bacteriology*, 177(4):998–1007.

[38] Trueba, F. J. and Woldringh, C. L. (1980). Changes in cell diameter during the division cycle of Escherichia coli. *Journal of Bacteriology*, 142(3):869.

[39] Turner, R. D., Mesnage, S., Hobbs, J. K., and Foster, S. J. (2018). Molecular imaging of glycan chains couples cell-wall polysaccharide architecture to bacterial cell morphology. *Nature Communications*, 9:1263.

[40] Viveiros, M., Dupont, M., Rodrigues, L., Couto, I., Davin-Regli, A., Martins, M., Pagès, J.-M., and Amaral, L. (2007). Antibiotic Stress, Genetic Response and Altered Permeability of E. coli. *PLoS ONE*, 2(4):e365.

[41] Vollmer, W., Blanot, D., and De Pedro, M. A. (2008). Peptidoglycan structure and architecture. *FEMS Microbiology Reviews*, 32(2):149–167.

[42] Vollmer, W. and Höltje, J.-V. (2004). The Architecture of the Murein (Peptidoglycan) in Gram-Negative Bacteria: Vertical Scaffold or Horizontal Layer(s)? *Journal of Bacteriology*, 186(18):5978–5987.

[43] Wong, F. and Amir, A. (2019). Mechanics and Dynamics of Bacterial Cell Lysis. *Biophysical Journal*, 116(12):2378–2389.
