# Supplementary material for: Whole-cell modeling of E. coli colonies enables quantification of single-cell heterogeneity in antibiotic responses
Source: PLoS Comput Biol. 2023 Jun 16;19(6):e1011232. doi: 10.1371/journal.pcbi.1011232 (PMC10310025; doi:10.1371/journal.pcbi.1011232)
Supplement: Attachment — Submitted filename: wcEcoli Colonies PLoS CB response.pdf [file pcbi.1011232.s013.pdf]

Overall comments

We were grateful for these reviews, which led us to the conclusions that our initial model lacked a deep integration of antibiotic response mechanisms with the whole-cell model and that nitrocefin was not a clinically or biologically relevant choice for an investigation of the antibiotic response. Addressing these concerns required a 18-month overhaul of the entire project in which we first re-implemented the entire whole cell *E. coli* model in Vivarium, confirmed that this newly migrated model mirrored previous outputs, and implemented mechanistic responses to two new antibiotics - ampicillin and tetracycline - with different mechanisms of action. Our new findings are essentially different, better validated, and deeper in insight. We couldn't thank the reviewers enough for making these suggestions, as although addressing their concerns was a significant undertaking, doing so has allowed us to craft a much stronger paper than before.

Reviewer #1:

The authors developed a software, Vivarium, that integrates a whole-cell model (WCM) and agent based model (ABM). This is the first agent-based multiscale model that simulates whole colonies of E. Coli. While whole-cell models focus on single cells, its utility for E. Coli is diminished as many behaviors of E. Coli are understood in the context of many interacting as a group, as in biofilms. Thus integrating WCMs and ABMs, the latter can simulate population behaviors that emerge from interactions of individual agents (or cells or bacteria), could address this gap. The authors' developed a unified model that simulates independent cells interacting in a shared spatial environment and links individual protein expressions to population-level phenotype. The model was used to demonstrate antibiotic resistance and simulated the colony's response to antibiotics.

In the model, the agents do not interact directly, but rather the interactions are mediated by changes in the environment, notably through multibody physics, including diffusion. The concentrations of molecules exchanged are through the environment as well. Using this model to investigate response to antibiotics, the authors' model indicate that variation in the expression level of the betalactamase AmpC, and not of the multi-drug efflux pump AcrAB-TolC, was the key mechanistic driver of survival of E. Coli in the presence of nitrocefin.

Overall, this is a novel approach, however, could the authors model a more commonly used antibiotic against E Coli (for example, doxycycline, ciprofloxacin, or nitrofurantoin) for which the antibiotic resistance is well characterized, which could further demonstrate the breadth of this model.

> We thank the reviewer for the suggestion, which we took very seriously in this major revision of our work. In particular, we have now removed nitrocefin from our study, in favor of two more commonly used antibiotics: tetracycline and ampicillin. These were selected for their differing modes of action: tetracycline is a bacteriostatic antibiotic that inhibits growth while ampicillin is a bactericidal antibiotic that kills cells outright. We have made sure to highlight the significant differences between the effects of these antibiotics

in the main text, lending stronger credence to our claims regarding the generalizability of our model. Furthermore, we have replaced the previous concentration-based threshold for cell death with more biologically accurate mechanisms of antibiotic action. These new mechanisms include tetracycline binding to ribosomes and consequent inhibition of protein synthesis, a tetracycline-induced gene regulatory program, ampicillin inactivation of cell wall synthesis machinery, and a coarse-grained model of the cell wall and lysis. The addition of these new mechanisms was made possible by a substantial rewrite of the whole-cell model to be much more deeply integrated with Vivarium. With this expanded model, we were able to derive more mechanistically-driven insights about *E. coli*'s response to two antibiotics, better demonstrating the value of our multi-scale approach.

Reviewer #2:

This study builds upon the whole cell Ecoli model (WCM) previously built by the authors and expands it to the population scale. The WCM model simulates individual Ecoli cells with high complexity. In stark contrast, their approach to model antibiotic response is extremely rudimentary and oversimplified. The insights from their population model are trivial and a direct consequence of their assumptions. The novel contribution here is the expansion to a whole colony model and application to antibiotic resistance. These ideas are not well developed unfortunately.

We thank the reviewer for encouraging us to develop a more detailed model of *E. coli*'s response to antibiotics. To fully leverage the complexity afforded by our WCM, we completely re-implemented the model within Vivarium, greatly improving the degree of integration between single cells and the broader colony simulation. This allowed us to include many well-established molecular mechanisms in our reworked model of the beta-lactam response, such as the inhibition of penicillin-binding proteins. For example, using parameters derived from a review of existing literature, our new cell wall sub-model mechanistically bridges the biochemical inhibition of murein synthesis and the biophysical process of cell wall rupture and lysis, a link that, as noted by the reviewer, was completely absent from our original model. Further inspired by the reviewer's comments, we expanded our modeling efforts to also include key mechanisms for *E. coli*'s response to an antibiotic with an entirely different mechanism of action, tetracycline. The resulting colony-scale model now has a much more direct connection between antibiotic response at the molecular level, and cell- to population- level effects. We do not believe that any of our new major findings come from obvious first assumptions. In sum, these efforts have transformed the paper, and we once again thank the reviewer for making the necessity of these efforts apparent.

Antibiotic resistance involves numerous transcriptional and metabolic effects. While we dont expect all details of antibiotic response to be modeled, well established mechanisms that are critical to antibiotic action should not be ignored. With the whole cell model they were in a good position to simulate the complexity, but unfortunately that's not the case here.

We have added new mechanistic sub-models for major known components of *E. coli's* response to tetracycline and ampicillin. We describe these mechanisms in detail in our new manuscript (see "*Tetracycline uniformly inhibits growth of simulated colonies in a dose-dependent manner*" and "*Ampicillin selectively kills simulated cells with low beta-lactamase concentrations*") and in our Supplementary Materials (see "Antibiotic response model"). For tetracycline, new mechanistic sub-models included the inactivation of ribosomes and induction of a gene regulatory program. For ampicillin, we modeled inactivation of cell wall synthesizing enzymes and the consequent damage to the cell wall leading to lysis.

The way the authors model antibiotic treatment is oversimplified. In the model, the drug kills the cells when it reaches an arbitrary threshold chosen by the authors. The primary targets of many of the antibiotics are well established. It should be straightforward to model the drug effect explicitly by inhibiting cell wall enzymes. Prior studies, for example from the Palsson lab, have used FBA models to simulate inhibition of antibiotic targets. The authors could easily build upon that.

We thank the reviewer for pointing out this limitation of our original work. In the many months since our original submission, we have significantly improved the level of detail contained within our model of antibiotic action. Rather than using a threshold for internal antibiotic concentration to determine death, we have now implemented a model for cell wall construction and are able to mechanistically simulate the consequences of cell wall synthesis inhibition by ampicillin, up to and including cell lysis upon development of a critical defect. Similarly, we have added new submodels for tetracycline that capture key mechanisms of action, such as protein synthesis inhibition via ribosome binding and induced gene regulation via the transcriptional activator MarA.

The authors conclude from their modeling that "the AcrAB-TolC concentration had little effect" on antibiotic response. This is incorrect and goes against several known observations. For example, how can they explain the fact that efflux pump inhibitors are synergistic with many antibiotics and are effective? Deletion of acrAB genes greatly increases sensitivity to antibiotics in genome-wide knockout screens. Overall, the model is too simplistic a model to make such a claim.

We thank the reviewer for this comment. In our revamped paper, we have added many new figures dedicated to validating our model against prior published results (Fig. 2C-D, S2, S3A, 4N). We have also provided a more thorough description of every new sub-model in the Supplementary Materials, including sources and/or derivation methods for all key parameters. As a result of this work, we now feel much greater confidence in our model's consistency with known observations.

In regards to the simplicity of our original model, we have completely reworked the beta-lactam response to include meaningful biological mechanisms. This new model uses parameters from literature to simulate the impact of ampicillin on cell wall integrity

on a molecular level, a great leap forward from our previous threshold for determining cell death.

Lastly, we appreciate the reviewer for pointing out the ambiguity of our phrasing regarding our findings related to AcrAB-TolC. In our new manuscript, we place a much greater emphasis on cell-cell heterogeneity in expression as opposed to raw expression values. We found that the natural heterogeneity in AcrAB-TolC concentration among cells in our colonies could not account for differences in cell survival during periods of lethal ampicillin exposure. At the same time, in our tetracycline simulations, we observed that cells with lower average AcrAB-TolC concentrations had higher average cytoplasmic tetracycline concentrations, in agreement with prior findings that AcrAB-TolC does indeed play an active role in the antibiotic response.

The authors state in the discussion (line 370-74) that many of the kinetic parameters related to beta lactamase and porin transcription, expression, and activity are unknown and could be inaccurate. The authors should report these parameters and do some type of sensitivity analysis of their results. If most parameters are unknown, maybe their model is not set up to answer this type of question.

We have replaced nitrocefin with ampicillin, and were able to draw on more known parameters in the creation of the new sub-model for the beta-lactam response. Unlike with nitrocefin, our model yielded an internal ampicillin concentration (1.27 μM) that was much closer to experimentally derived estimates (1.7 μM) (1). This suggests that the new parameters are more accurate. The fact that the time scales of lysis produced by our model are close to experimental measurements (Fig. 4N) gives us further confidence in the accuracy of our new model.

In the abstract and discussion, the authors report that they use a novel software called vivarium, which is misleading, as vivarium is not presented here but is reported in a different related manuscript. The authors cannot claim novelty in two separate papers.

We agree with the reviewer's observation and have replaced claims about the novelty of Vivarium with the literature citation.

There is no validation per se for the whole colony model. The only 'validation' reported in line 202, which they state "deepened our confidence in the simulations" is trivial. The authors found that the model produced higher growth in aerobic vs anaerobic. This has nothing to do with the colony model. Even a simple FBA model could have done this.

We agree with the reviewer's observation and have put more focus on validation in the new manuscript. First, to validate that our Vivarium-based rewrite of the whole-cell model reproduced experimentally measured behavior in basal glucose conditions, we added new supplementary figures which show that average protein counts and fluxes through reactions in central carbon metabolism in our simulations compare favorably with corresponding values from literature (Fig. S2). We also noted that the average doubling

> time of our cells was close to experimental measurements and therefore yielded the expected amount of colony growth over time (Fig. S3A). Lastly, we updated the constant for glucose diffusion in accordance with more recent data (2) and no longer observed the formation of significant glucose gradients in even our largest colonies (~256 cells). This is in line with a prior study showing that oxygen depletion only occurs in much thicker and denser biofilms than our simulated microcolonies (3). This comparison was added to our main text (see "*Simulated colonies exhibit phenotypic heterogeneity*"). Taken together, this gave us confidence that our model was able to accurately capture biologically relevant phenomena across all modeled spatial scales.

How is AmpC induced and regulated in response to antibiotics? How long is the time delay for induction? It appears to be constitutively expressed. I am surprised ampC is even expressed in their model. It is usually expressed in clinical strains. The model they are using is not a clinical strain or at least it wasn't mentioned in the manuscript. AmpC expression is usually low but inducible in response to β-lactam exposure.

> We have now double-checked our assertions, and found that expression of *ampC* is, indeed, considered noninducible and low in most strains of *E. coli* (4,5). In particular, one study found that non-clinical MG1655 K-12 *E. coli*, the strain used to parameterize gene expression in our model, express, on average, 132 AmpC monomers per generation when growing in MOPS minimal media with glucose (6), close to our basal glucose media. An additional source found that about 7e-5% of the total mRNAs in NCM3722 K-12 cells are transcripts of the *ampC* gene (7). Upon converting these measurements to their concentration equivalents, we found that the expression of *ampC* mRNA and AmpC monomers is consistent with prior measurements (Fig. 2C-D, colored pins on right side of timeseries plots).

What are the induction times for ampc and tolc?

> While we were not able to find evidence that *ampC* expression was induced in the presence of tetracycline or ampicillin, we have implemented a mechanism by which *E. coli* can upregulate the transcription probability of *tolC* in response to tetracycline. Interestingly, we found that the programmed level of upregulation, while close to experimental measurements (Fig. S6B, blue), was not sufficient to counteract the effects of protein synthesis inhibition, causing AcrAB-TolC concentration to slowly decrease over time. Additionally, we dedicated a portion of our tetracycline results (Fig. 3I-K) to an analysis of the timescales for OmpF porin downregulation. We found that while the average *ompF* mRNA concentration declined by half in about 5 minutes, average OmpF protein monomer concentration only halved after over 1.5 hours.

How was protein specific heterogeneity parameters determined in the model? How/why is Ampc more noisy than Acrab? The reason for this, and if it was intentionally programmed, should be discussed.

We appreciate the reviewer's question about the source of expression heterogeneity in our model. We have made heterogeneity a much greater focus throughout the main text and added explanations for the phenomenon of subgenerational expression, which describes a pattern of expression in which genes are, on average, transcribed less than once per generation (see Figure 1 and sections "*Heterogeneity and interaction effects motivate whole-colony model*" and "*Simulated colonies exhibit phenotypic heterogeneity*"). In our original release of the whole-cell model, when fitting transcription probabilities for individual genes to match experimental RNA-seq measurements, certain genes were assigned such low expression probabilities that they could grow for one or more generations without a single transcription event (8). In this paper, we showed that *ampC* was among the genes that exhibited subgenerational expression while *tolC* was not, which helps explain, in part, why the concentration of AmpC tended to be much more variable than that of AcrAB-TolC.

Is there a cost for producing beta lactamase? If not, why is that the case?

Since our model has such low, non-inducible expression of beta-lactamase, we did not expect to find any significant cost to beta-lactamase production. Indeed, there was negligible correlation between AmpC concentration and growth rate (data not shown).

I think there would be some type of interaction effect between porins and belactamase activity, but I'm surprised they did not see anything.

Re-implementing the WCM in Vivarium allowed us to update our model of diffusion to respond to differences in OmpF porin concentrations, such that cells with no OmpF had antibiotic permeability equivalent to that measured experimentally in OmpF knockout cells. We focused on OmpF specifically because prior work has suggested that it is the primary channel for diffusion of our chosen antibiotics, with knockouts of other porins yielding no significant increases in resistance to either tetracycline or ampicillin (9).

Nitrocefin is technically not an antibiotic. It is an indicator dye for beta lactamase detection.

This is a very good point. In response to comments by both Reviewer 1 and Reviewer 2, we have replaced nitrocefin with ampicillin and tetracycline, two more commonly used antibiotics.

Their model currently treats the drug treatment as a bacteriostatic effect, but betalactams are bactericidal. The dead cells should be removed from simulation.

We have changed the beta-lactam response to trigger lysis, with cells spilling out their internal contents and being removed from the simulation upon death. Additionally, we allowed extracellular beta-lactamases to continue to hydrolyze beta-lactams in the environment, as would be the case following real cell lysis.

Why do dead cells take up space?

With the new cell lysis mechanism for ampicillin, dead cells are now removed from the simulation after spilling their contents into the environment. The response to tetracycline, however, is bacteriostatic, and no cells are removed. We describe these mechanisms in our manuscript under "*Ampicillin selectively kills simulated cells with low beta-lactamase concentrations*" and "*Tetracycline uniformly inhibits growth of simulated colonies in a dose-dependent manner*", and in our Supplementary Materials under "Antibiotic response model" (particularly in the subsections "Cell wall growth, division and lysis" and "Tetracycline binding to ribosomes").

Not sure how they got the MIC value for nitrocefin. The original nitrocefin article reports 64 ug/ml

We chose to replace nitrocefin with ampicillin in accordance with suggestions by both reviewers. However, for the sake of completeness, we note that in the previous manuscript we simply converted the MIC from µg/mL to mM (molecular weight from PubChem):

$$\frac{64\ \mu g}{mL} * \frac{g}{10^6\ \mu g} * \frac{mol}{516.5\ g} * \frac{10^3\ mmol}{mol} * \frac{10^3 mL}{L} = 0.1239\ mM$$

The authors should take an unbiased approach and compare all the molecular properties of alive and dead cells.

We thank the reviewer for the suggestion to broaden our analysis of antibiotic-treated cells. We used our newly expanded model to compare molecular properties of cells before and after antibiotic exposure. For tetracycline, this revealed that active ribosome and OmpF porin counts went down as did single-cell and whole-colony growth rate, as expected (see Fig. 3D, H-K, and M). More surprisingly, we found that the concentration of active RNA polymerases and, as a result, mRNA mass, experienced dramatic decreases at the MIC. For ampicillin, this revealed that variability in AmpC was, as before, the greatest differentiator between live and dead cells, more so than many other proteins relevant to our implemented antibiotic response mechanisms (see Fig. 4L, M).

The authors describe the implementation of the physics problem related to forces acting on cells. But no results are described from this analysis. What did we learn from the physics problem?

We appreciate the reviewer for the suggestion to take a closer look at the results derived from incorporating a physics model (first described (10)) into our colony simulations. Upon doing so, we found that there was a surprisingly noisy correlation between phylogenetic relatedness and physical proximity of cells (see "*Simulated colonies exhibit phenotypic heterogeneity*"), a direct consequence of the jostling between neighboring cells that was simulated by our new multibody physics sub-model (described under "*Spatial environment model*" in the Supplementary Materials). In the *Discussion* section of our new manuscript, we have added a sentence describing an interesting implication this might have for microscopy experiments: physical proximity alone might not be a strong proxy for phylogenetic relatedness.

The authors refer to the antibiotic resistance regulator, marA, in the introduction and discussion but never use it in the paper.

> We appreciate the reviewer for pointing us towards MarA for our revisions. After reviewing the literature, we decided to add sub-models for an entirely different type of antibiotic that was not discussed in our original submission, tetracycline. Specifically, we added a sub-model for tetracycline-induced gene regulation that is orchestrated entirely by MarA and explored how the resultant changes in gene expression affected cellular responses to tetracycline over time. The mechanism and its results are described in the section "*Tetracycline uniformly inhibits growth of simulated colonies in a dose-dependent manner*", and details of the model implementation are contained in our Supplementary Materials in the "Antibiotic response model" section.

line 120: Which molecules from WCM are reported to Vivarium? Why not all? What is the rationale for selecting a small set of genes/features?

> We re-implemented the WCM within Vivarium, allowing us to save any and all data tracked by the model. Because the total output from simulations quickly grew to the order of terabytes, we chose to retrieve and analyze specific information that we felt would be most relevant to our mechanisms, including counts of all protein monomers, counts of mRNAs, antibiotic concentrations, cell wall metadata (e.g. maximum hole size, porosity, etc.), and cell submasses (e.g. total, protein, mRNA, etc.).

line 178: The authors report variation in glucose levels. What about other nutrients and secreted metabolites? Do they change? that could be reported in a heatmap and correlated with antibiotic response.

> We thank the author for the suggestion to revisit the variation in environmental glucose levels. Upon doing so, we found that we had used an incorrect diffusion coefficient for glucose in our model of the spatial environment. After updating the glucose diffusion coefficient (source provided under "*Spatial environment model*" in the Supplementary Materials), we found that there was no longer significant variation in environmental glucose levels when growing colonies of the size that we were able to simulate. Accordingly, we have removed this section from the main text.
>
> With regard to changes in nutrients and metabolites, we believe that this could be a very fruitful and interesting area for future investigation. At present, we have centered our paper primarily around the influence of heterogeneity in gene and protein expression on the antibiotic response. However, we are very interested in the idea of taking a closer look at metabolic changes in response to antibiotics in the near future.

References Cited

1. Kojima S, Nikaido H. Permeation rates of penicillins indicate that Escherichia coli porins function principally as nonspecific channels. Proc Natl Acad Sci U S A. 2013 Jul

9;110(28):E2629–34.
2. Suhaimi H, Wang S, Das DB. Glucose diffusivity in cell culture medium. Chem Eng J. 2015 Jun 1;269:323–7.
3. Zhang TC, Fu YC, Bishop PL. Competition for substrate and space in biofilms. Water Environ Res. 1995;67(6):992–1003.
4. Livermore DM. beta-Lactamases in laboratory and clinical resistance. Clin Microbiol Rev. 1995 Oct;8(4):557–84.
5. Jacoby GA. AmpC β-Lactamases. Clin Microbiol Rev. 2009 Jan;22(1):161–82.
6. Li GW, Burkhardt D, Gross C, Weissman JS. Quantifying Absolute Protein Synthesis Rates Reveals Principles Underlying Allocation of Cellular Resources. Cell. 2014 Apr 24;157(3):624–35.
7. Balakrishnan R, Mori M, Segota I, Zhang Z, Aebersold R, Ludwig C, et al. Principles of gene regulation quantitatively connect DNA to RNA and proteins in bacteria. Science. 2022 Dec 9;378(6624):eabk2066.
8. Macklin DN, Ahn-Horst TA, Choi H, Ruggero NA, Carrera J, Mason JC, et al. Simultaneous cross-evaluation of heterogeneous E. coli datasets via mechanistic simulation. Science. 2020 Jul 24;369(6502):eaav3751.
9. Choi U, Lee CR. Distinct Roles of Outer Membrane Porins in Antibiotic Resistance and Membrane Integrity in Escherichia coli. Front Microbiol [Internet]. 2019 [cited 2023 Mar 3];10. Available from: https://www.frontiersin.org/articles/10.3389/fmicb.2019.00953
10. Agmon E, Spangler RK, Skalnik CJ, Poole W, Peirce SM, Morrison JH, et al. Vivarium: an interface and engine for integrative multiscale modeling in computational biology. Bioinforma Oxf Engl. 2022 Mar 28;38(7):1972–9.
